# A real-world study of Trifluridine/Tipiracil (TAS-102) combined with bevacizumab as the late-line treatment of metastatic colorectal cancer

**DOI:** 10.1007/s12672-026-04459-6

**Published:** 2026-02-01

**Authors:** Shaocheng Zeng, Huangying Deng, Hanzhi Dong, Chunye Huang, Ruiwen Ruan, Xiaofeng Dai, Jianping Xiong, Jun Deng, Yangyang Yao

**Affiliations:** 1 Department of Oncology, The First Affiliated Hospital, Jiangxi Medical College, Nanchang University, Nanchang, 330006 Jiangxi China; 2 Jiangxi Key Laboratory for Individual Cancer Therapy, Nanchang, 330006 Jiangxi China; 3 Department of Medical Oncology, Jiangxi Cancer Hospital, The Second Affiliated Hospital of Nanchang Medical College, Jiangxi Clinical Research Center for Cancer, Nanchang, 330029 Jiangxi China

**Keywords:** Trifluridine/Tipiracil, Bevacizumab, Metastatic colorectal cancer, Prognostic analysis

## Abstract

**Background:**

Trifluridine/Tipiracil (TAS-102) is an effective agent for the late-line treatment of metastatic colorectal cancer (mCRC). Combining TAS-102 with bevacizumab improves outcomes but may increase adverse events. We conducted a real-world, retrospective, exploratory comparison of two dosing schedules (bi-weekly vs. four-weekly) to describe efficacy, safety, and potential molecular and clinical correlates.

**Methods:**

We analyzed patients with mCRC who were treated with TAS-102 in combination with bevacizumab as late-line therapy from January 2020 to February 2023. Regimen assignment followed physician-patient shared decision-making based on clinical factors and local practice changes after emerging evidence, not randomization. Endpoints included progression-free survival (PFS), overall survival (OS), adverse events (AEs). Analyses were exploratory and hypothesis-generating, with multivariable Cox models for selected covariates.

**Results:**

A total of 92 patients were enrolled in this study. Median PFS was 3.2 months (bi-weekly) vs. 3.7 months (four-weekly), and median OS was 10.0 vs. 9.3 months, with no statistically significant differences. KRAS mutation was associated with inferior OS (7.7 vs. 11.8 months; *P* = 0.018), whereas TP53 was not. Eastern Cooperative Oncology Group performance status (ECOG-PS) = 2 independently predicted shorter PFS and OS; prior bevacizumab exposure correlated with shorter PFS but not OS. Common adverse events in patients were neutropenia (63.0%), leukopenia (67.0%), anemia (44.6%), malaise (55.4%), nausea (45.7%), anorexia (31.5%), and diarrhea (23.9%).

**Conclusion:**

In this retrospective, real-world study, the two regimens demonstrated comparable disease control, and the bi-weekly regimen appeared to be better tolerated, representing a reasonable potential alternative. Nevertheless, these findings should be interpreted as exploratory, and future prospective studies are warranted.

## Introduction

Colorectal cancer (CRC) is among the most common malignancies worldwide, with approximately 2 million new cases in 2020 and accounting for about 10% of all cancers and cancer-related deaths each year [1]. The OS of patients with metastatic colorectal cancer (mCRC) has been improving over the past 20 years, with the median now reaching more than 30 months [2–5]. First-line therapy for mCRC include chemotherapeutic agents (e.g., fluorouracil, irinotecan, and oxaliplatin) in combination with biologically targeted agents such as cetuximab and bevacizumab [6, 7]. Nevertheless, most patients experience disease progression. Late-line options include TAS-102, targeted therapy (e.g., regorafenib, fruquintinib), and immunotherapy (e.g., nivolumab, pembrolizumab), with TAS-102 increasingly used in this setting.

Trifluridine/tipiracil (TAS-102), approved in Japan in 2014 for late-line CRC, remains effective in fluorouracil-resistant disease [8]. Tipiracil inhibits thymidine phosphorylase, limiting trifluridine degradation and enhancing its bioavailability, thereby promoting DNA damage and tumor cell death [9–11]. In mCRC, TAS-102 improved OS versus placebo in RECOURSE (7.1 months; 95% CI, 6.5–7.8; vs. 5.3 months; 95% CI, 4.6-6.0) and in TERRA (7.8 months; 95% CI, 7.1–8.8; vs. 7.1 months; 95% CI, 5.9–8.2) [12]. However, several reports suggest OS comparable to regorafenib [13, 14], motivating combinations with targeted agents such as bevacizumab.

Bevacizumab inhibits tumor angiogenesis, growth, and metastasis by inhibiting the physiological functions of VEGF, such as vascular permeability, proliferation, and endothelial cell migration and survival [15–17]. By normalizing tumor vasculature and reducing interstitial pressure, bevacizumab may improve drug delivery and increase intratumoral concentrations of cytotoxic agents [18]. This results in an increased concentration of chemotherapeutic agents within the tumor.

Recent studies have evaluated TAS-102 plus bevacizumab as late-line therapy for mCRC. The C-TASK FORCE phase I/II trial reported a 16-week PFS of 42.9% (80% CI, 27.8–59.0), outperforming TAS-102 monotherapy [19]. The further TAS-CC3 study showed mPFS of 4.5 months (95% CI, 1.8–7.1) and a mOS of 9.2 months (95% CI, 5.5–12.8), which were comparable to the results of the C-TASK FORCE study [20]. Against this backdrop, we conducted a real-world, retrospective analysis comparing bi-weekly and four-weekly schedules in routine practice. Our primary objective was to describe efficacy and safety signals; a secondary objective was to explore molecular and clinical correlates of outcome.

## Methods

### Patients

This retrospective, real-world study included consecutive adults with mCRC who received TAS-102 plus bevacizumab after failure of at least two prior systemic lines from January 2020 to February 2023. All patients were diagnosed with mCRC by histopathology or cytopathology. In addition, all patients had at least 1 measurable lesion by Response Evaluation Criteria in Solid Tumors Version 1.1 (RECIST1.1). Key exclusions were insufficient clinical records and, for efficacy analyses, patients who did not complete at least two cycles (to ensure adequate exposure and at least one tumor assessment). This study was approved by the Ethics Committee of the First Affiliated Hospital of Nanchang University (IIT2024163).

### Treatment

Patients received either a bi-weekly or a four-weekly TAS-102 schedule combined with bevacizumab. Regimen selection was not randomized, it reflected evolving local practice and physician-patient shared decision-making.

1. TAS-102: The recommended dose of 70 mg/m² per day is divided into two daily doses to be taken orally within 1 h after breakfast and dinner. Dose adjustment were determined by the clinician. Four-weekly regimen: start on day 1 of each cycle, take 5 consecutive days, discontinue for 2 days, take another 5 days, discontinue for 16 days, 28 days for 1 cycle. Bi-weekly regimen: 5 consecutive days, 9 days off, 14 days for 1 cycle.
2. Bevacizumab: Intravenous injection on day 1 of each cycle at a dose of 5 mg/kg calculated once every 2 weeks.

### Molecular testing

The KRAS/NRAS/TP53 status of the patients was analyzed using next-generation sequencing (NGS) at the participating institution. Testing platforms varied by calendar period and tissue availability.

### End points and assessments

The primary endpoint was progression-free survival (PFS), measured from treatment initiation to disease progression or study cutoff. Overall survival (OS) served as the secondary endpoint. Objective response rate (ORR) was defined as the proportion of patients achieving complete or partial response, while disease control rate (DCR) included patients with stable disease, partial response, or complete response. Baseline tumor evaluation used the most recent pre-treatment imaging (CT, MRI, or PET/CT). Follow-up imaging was conducted every two treatment cycles, with responses assessed per RECIST 1.1. Laboratory values and tumor markers were recorded at baseline and before each cycle. Adverse events were graded according to the Common Terminology Criteria for Adverse Events version 5.0 (CTCAE v5.0).

### Statistical analysis

Baseline characteristics were summarized descriptively. Group comparisons utilized chi-square or Fisher’s exact tests for categorical variables and Wilcoxon rank-sum tests for continuous variables. Progression-free survival (PFS) and overall survival (OS) were estimated by the Kaplan-Meier method and compared using log-rank tests. Multivariable Cox proportional hazards models were constructed to adjust for potential confounders, including age, ECOG-PS, number of prior lines, prior bevacizumab exposure, primary tumor sidedness, and KRAS/NRAS/TP53 status where available. Given the substantial imbalance in sample size between regimens and the limited number of events in the bi-weekly group, we refrained from propensity score weighting to avoid unstable estimates. All efficacy and safety comparisons between dosing schedules are therefore exploratory and hypothesis-generating. Two-sided P values < 0.05 were considered statistically significant without adjustment for multiplicity.

## Results

### Patients

**Fig. 1:**
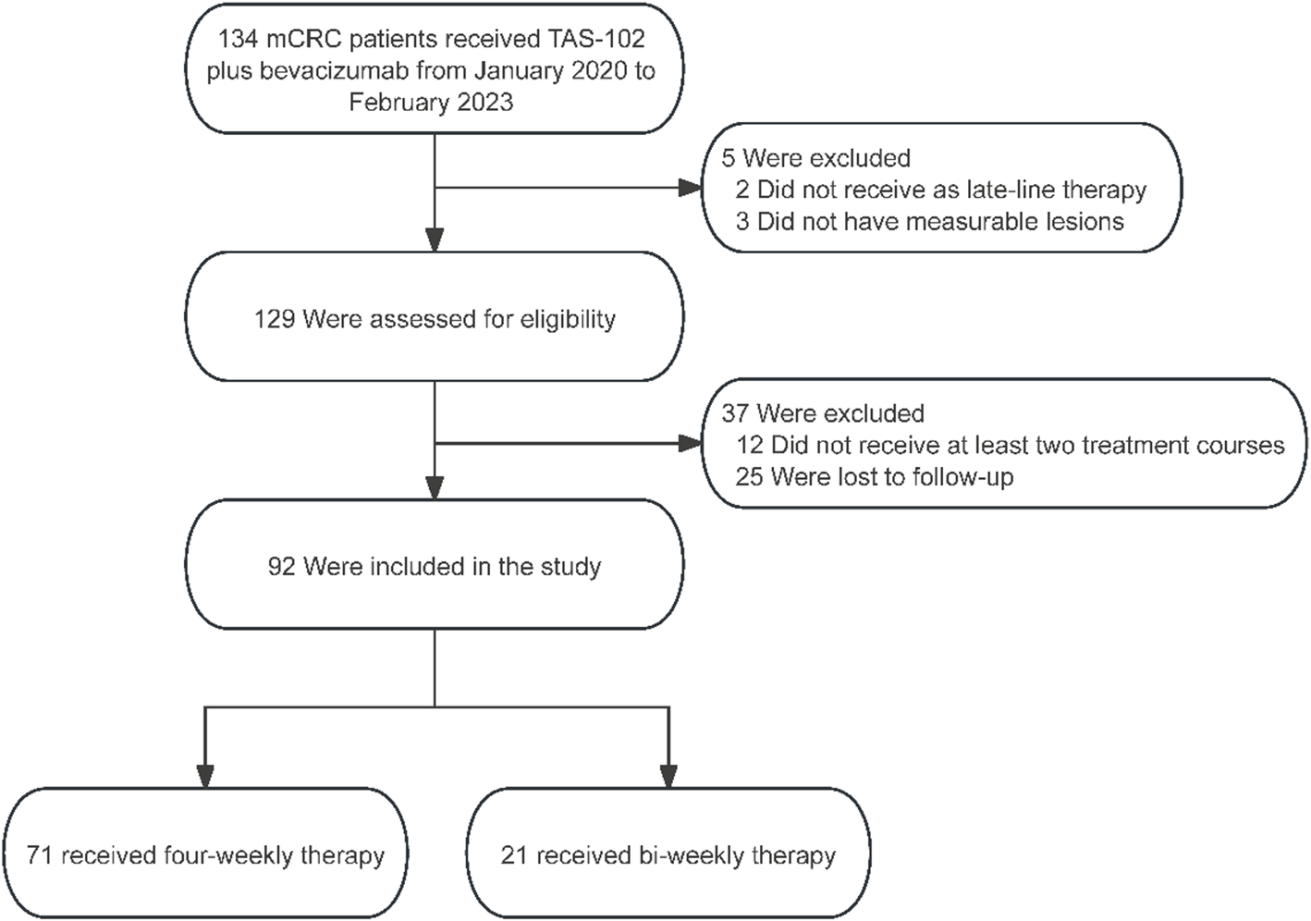
Flow chart of this study

From January 2020 to February 2023, 134 patients were screened (Fig. 1); 42 were excluded, leaving 92 patients with mCRC for analysis. The treatment groups were imbalanced: 21 patients received the bi-weekly regimen and 71 received the four-weekly regimen (Fig. 1). Most patients were male (57/92, 61.9%) and younger than 65 years (67/92, 72.8%); the mean age was 55.6 years. Sixty of 92 patients (65.2%) had a normal BMI, and 79/92 (85.8%) had an ECOG PS of 0–1.

Baseline characteristics are summarized in Table 1. No significant between-group differences were observed across clinical variables (all *P* > 0.05). Overall, the primary tumor site was the colon in 57 patients (62.0%); most had TNM stage IVB disease (64/92, 69.5%). The number of metastatic sites was ≤ 3 in 77 patients (83.7%), and liver metastases were present in 72 (78.3%). Regarding prior therapy, > 90% had received fluorouracil-based regimens, oxaliplatin, and irinotecan; 73 (79.3%) had undergone resection of the primary tumor. Molecular results (KRAS, NRAS, P53) were available for 63 patients (68.5%), and MSI status for 83 (90.2%).

**Table 1:** Clinical characteristics of patients and treatments

Characteristic	All patients(*n* = 92) *n*(%)	Bi-weekly (*n* = 21) *n*(%)	Four-weekly (*n* = 71) *n*(%)	*P* value
**Gender**				0.996
Male	57(61.9)	13(61.9)	44(61.9)	
Female	35(38.1)	8(38.1)	27(38.1)	
**Age(years)**				0.131
≥ 65	25(27.1)	3(14.2)	22(30.9)	
<65	67(72.8)	18(85.7)	49(69.0)	
Median(range)	57(26–87)	56(34–75)	57(26–87)	
**BMI**				0.268
≥ 24	16(17.3)	5(23.8)	11(15.4)	
18 ≤ BMI<24	60(65.2)	13(61.9)	47(66.2)	
<18	16(17.3)	3(14.2)	3(4.2)	
**ECOG-PS**				0.981
0–1	79(85.8)	18(85.7)	61(85.9)	
2	13(14.1)	3(14.2)	10(14.1)	
**Primary tumor site**				0.613
Rectum	57(62.0)	14(66.7)	43(60.6)	
Colon	35(38.0)	7(33.3)	28(39.4)	
**TNM**				0.113
ⅣA	9(9.7)	4(19.1)	5(7.0)	
ⅣB	64(69.5)	11(52.3)	53(74.6)	
ⅣC	19(20.6)	6(28.5)	13(18.3)	
**Number of metastatic sites**				0.289
> 3	15(16.3)	5(23.8)	10(14.1)	
≤ 3	77(83.7)	16(76.2)	61(85.9)	
**Liver metastasis**				0.793
Yes	72(78.3)	16(76.2)	56(78.9)	
No	20(21.7)	5(23.8)	15(21.1)	
**Previous treatment lines**				0.328
≤ 2	30(32.6)	5(23.8)	25(35.2)	
>2	62(67.3)	16(76.1)	46(64.7)	
**Previous treatment agents**				0.939
Fluorouracil	92(100.0)	21(100.0)	71(100.0)	
Oxaliplatin	83(90.2)	19(90.4)	64(90.1)	
Irinotecan	85(92.3)	21(100)	64(90.1)	
Bevacizumab	73(79.3)	19(90.4)	54(76)	
Cetuximab	26(28.2)	7(33.3)	19(26.7)	
Regorafenib	36(39.1)	9(42.8)	27(38.0)	
Fruquintinib	19(20.6)	2(9.5)	17(23.9)	
ICIs	39(42.3)	9(42.8)	30(42.2)	
**Previous surgery**				0.239
Primary tumor	73(79.3)	14(66.6)	59(83.1)	
Metastatic tumor	23(25)	2(9.5)	21(29.5)	
**KRAS**				0.149
Wild type	37(40.2)	11(52.3)	26(36.6)	
Mutant	26(28.2)	7(33.3)	19(26.7)	
Unknown	29(31.5)	3(14.2)	26(36.6)	
**NRAS**				0.154
Wild type	56(60.8)	16(76.1)	40(56.3)	
Mutant	7(7.6)	2(9.5)	5(7.0)	
Unknown	29(31.5)	3(14.2)	26(36.6)	
**P53**				0.074
Wild type	48(52.1)	12(57.1)	36(50.7)	
Mutant	15(16.3)	6(28.5)	9(12.6)	
Unknown	29(31.5)	3(14.2)	26(36.6)	
**BRAF**				0.117
Wild type	61(66.3)	17(80.9)	44(61.9)	
Mutant	2(2.1)	1(4.7)	1(1.4)	
Unknown	29(31.5)	3(14.2)	26(36.6)	
**MSI status**				0.819
MSS	80(86.9)	19(90.5)	61(85.9)	
MSI-L	1(1.0)	0(0.0)	1(1.4)	
MSI-H	2(2.1)	0(0.0)	2(2.8)	
Unknown	9(9.8)	2(9.5)	7(9.9)	

BMI, body mass index; ECOG-PS, Eastern Cooperative Oncology Group performance status; TNM, tumor node metastasis; MSI, microsatellite instability; MSI-H, high microsatellite instability; MSI-L, low microsatellite instability; MSS, stable microsatellite instability

### Efficacy

**Fig. 2:**
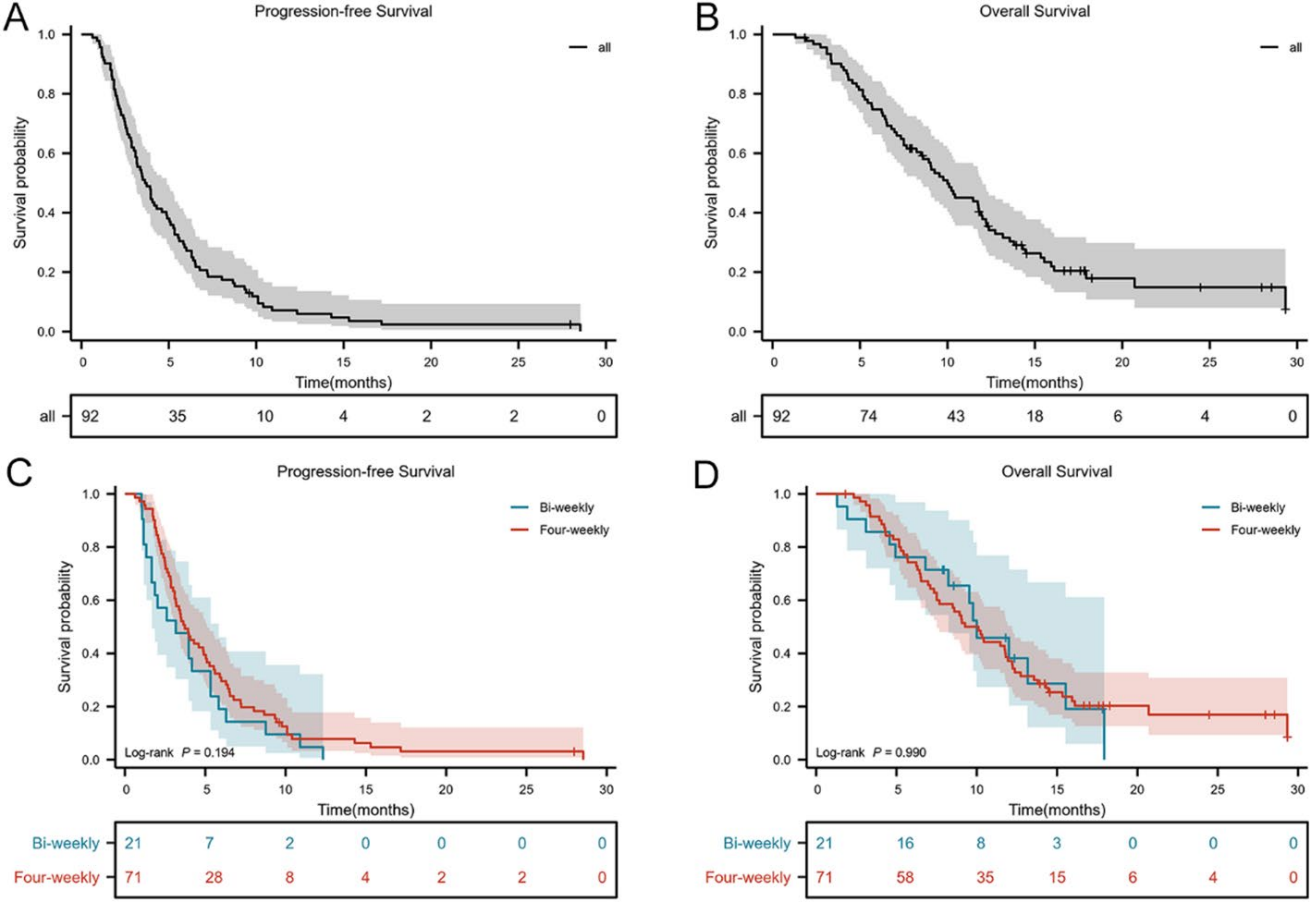
Kaplan-Meier curves of patients. A and B: PFS and OS of all patients; C and D: PFS and OS of bi-weekly and four-weekly group

At the data cutoff (January 10, 2024), 67 patients (72.8%) had died and 25 (27.2%) were alive; the median follow-up was 16.7 months. No complete responses (CR) were observed. The best overall response consisted of partial response (PR) in 3 patients, stable disease (SD) in 64, and progressive disease (PD) in 25, yielding an overall response rate (ORR) of 3.2% and a disease control rate (DCR) of 72.8% (Table 2). In the bi-weekly group, there were no PRs; 15 patients (71.4%) achieved SD and 6 (28.6%) had PD (ORR 0%; DCR 71.4%). In the four-weekly group, 3 patients (4.2%) achieved PR, 49 (69.0%) had SD, and 19 (26.8%) had PD (ORR 4.2%; DCR 73.2%). Between-group differences in ORR and DCR were not statistically significant (*P* = 0.338 and *P* = 0.870, respectively). For all patients, median PFS and median OS were 3.6 months (95% CI, 2.913–4.354) and 10.0 months (95% CI, 7.860–12.140), respectively (Fig. 2A and B). In the bi-weekly group, median PFS and OS were 3.2 months (95% CI, 0.999–5.335) and 10.0 months (95% CI, 7.183–12.817); in the four-weekly group, they were 3.7 months (95% CI, 2.877–4.590) and 9.3 months (95% CI, 7.388–11.146), respectively. Differences in PFS and OS between the two regimens were not statistically significant (*P* = 0.194 and *P* = 0.990; Fig. 2C, D).

**Table 2:** Comparison of efficacy in patients

Best response	Total (*n* = 92)	Treatment		*P* value
		Bi-weekly (*n* = 21)	Four-weekly (*n* = 71)	
CR	0	0	0	–
PR	3	0	3	–
SD	64	15	49	–
PD	25	6	19	–
ORR	3(3.2%)	0(0.0%)	3(4.2%)	0.338
DCR	67(72.8%)	15(71.4%)	52(73.2%)	0.870

CR, complete response; PR, partial response; SD, stable disease; PD, progress disease; PFS, progression-free survival; OS, overall survival; ORR, objective response rate; DCR, disease control rate

### Biomarker analyses

**Fig. 3:**
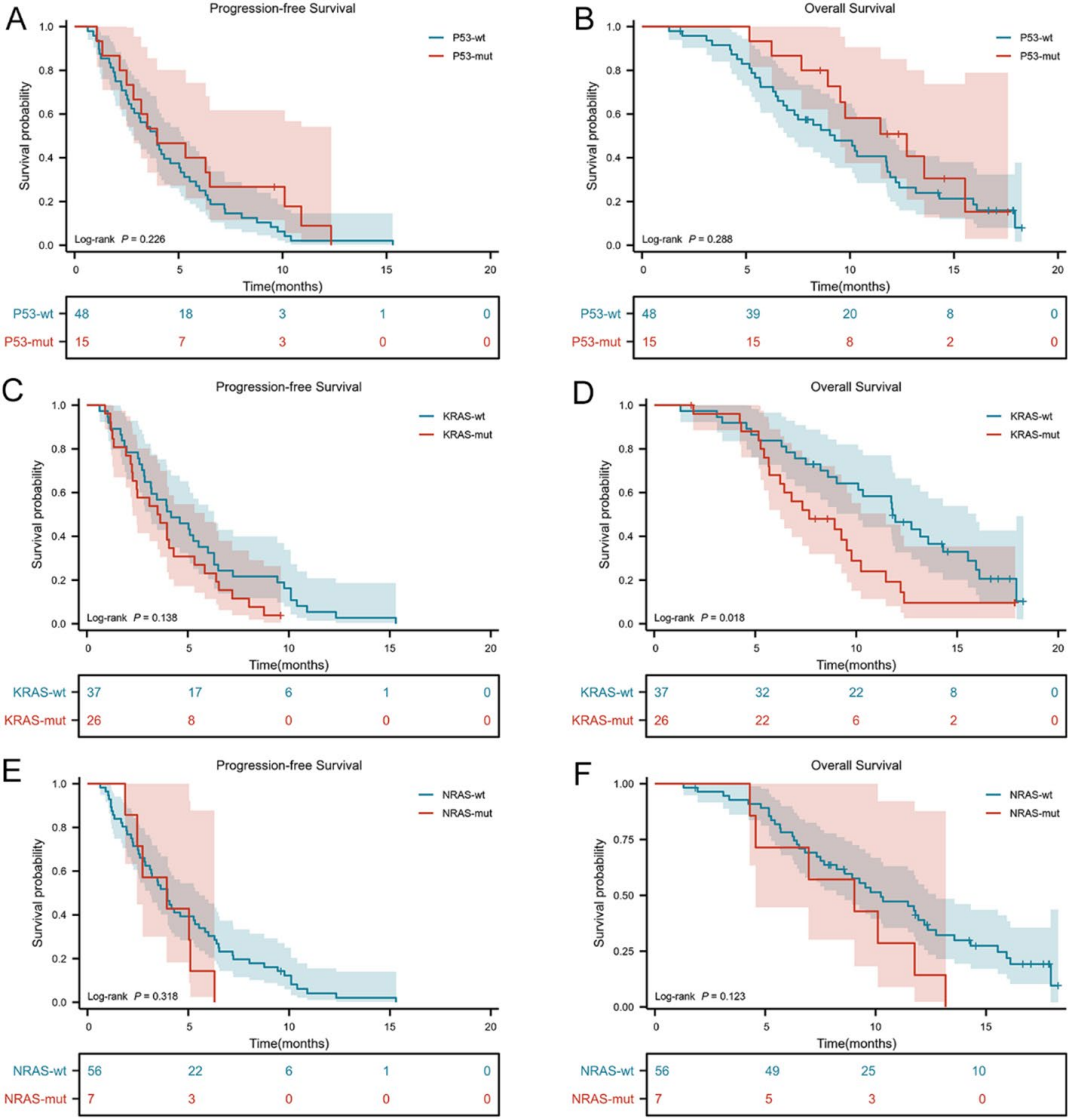
A and B PFS and OS in P53 wild-type and mutant patients; C and D: PFS and OS in KRAS wild-type and mutant patients; E and F: PFS and OS in NRAS wild-type and mutant patients. P53 wild-type, P53-wt; P53 mutant, P53-mut; KRAS wild-type, KRAS-wt; KRAS mutant, KRAS-mut; NRAS wild-type, NRAS-wt; NRAS mutant, NRAS-mut;

Molecular data were available for 63 patients (68.5%), grouped by P53, KRAS, and NRAS status (Fig. 3). ​Reasons for missing data included exhaustion of archival tissue samples, unavailability of test reports, or lack of testing due to financial constraints. For P53, median PFS and OS were 3.93 months (95% CI, 2.915–4.952) and 9.267 months (95% CI, 6.834–11.700) in P53-wt versus 3.98 months (95% CI, 1.274–6.660) and 12.733 months (95% CI, 8.061–17.406) in P53-mut, with no statistically significant differences (*P* = 0.226 and *P* = 0.286; Fig. 3A and B). For KRAS, median PFS was 4.2 months (95% CI, 2.300–6.034) in KRAS-wt versus 3.5 months (95% CI, 2.034–4.966) in KRAS-mut (*P* = 0.136; Fig. 3C), whereas median OS was 11.8 months (95% CI, 10.453–13.147) versus 7.7 months (95% CI, 4.403–10.931), respectively (*P* = 0.018; Fig. 3D). For NRAS, median PFS was 4.0 months (95% CI, 3.235–4.689) in wild type versus 3.9 months (95% CI, 3.302–4.631) in mutant, and median OS was 10.2 months (95% CI, 7.518–12.949) versus 9.0 months (95% CI, 3.730–14.337), without statistically significant differences (*P* = 0.318 and *P* = 0.123). Given the small number of NRAS‑mutant cases (*n* = 7), these analyses are underpowered and should be considered hypothesis‑generating.

### Exploratory analyses of clinical factors

Associations between clinical characteristics and outcomes are shown in Tables 3 and 4. In univariate analyses, shorter PFS was significantly associated with having ≥ 3 metastatic sites (HR, 2.001; 95% CI, 1.134–3.530; *P* = 0.017), prior bevacizumab exposure (HR, 1.751; 95% CI, 1.035–2.962; *P* = 0.037), and ECOG PS = 2 (HR, 2.777; 95% CI, 1.518–5.082; *P* < 0.001). No significant associations with PFS were observed for age, sex, primary site, liver metastasis, prior resection of the primary tumor, or number of prior lines. Variables with *P* < 0.10 in univariate analysis were entered into a multivariable Cox model; prior bevacizumab exposure (HR, 1.842; 95% CI, 1.077–3.151; *P* = 0.026) and ECOG PS = 2 (HR, 2.447; 95% CI, 1.166–5.341; *P* = 0.018) remained independently associated with shorter PFS.

**Table 3:**
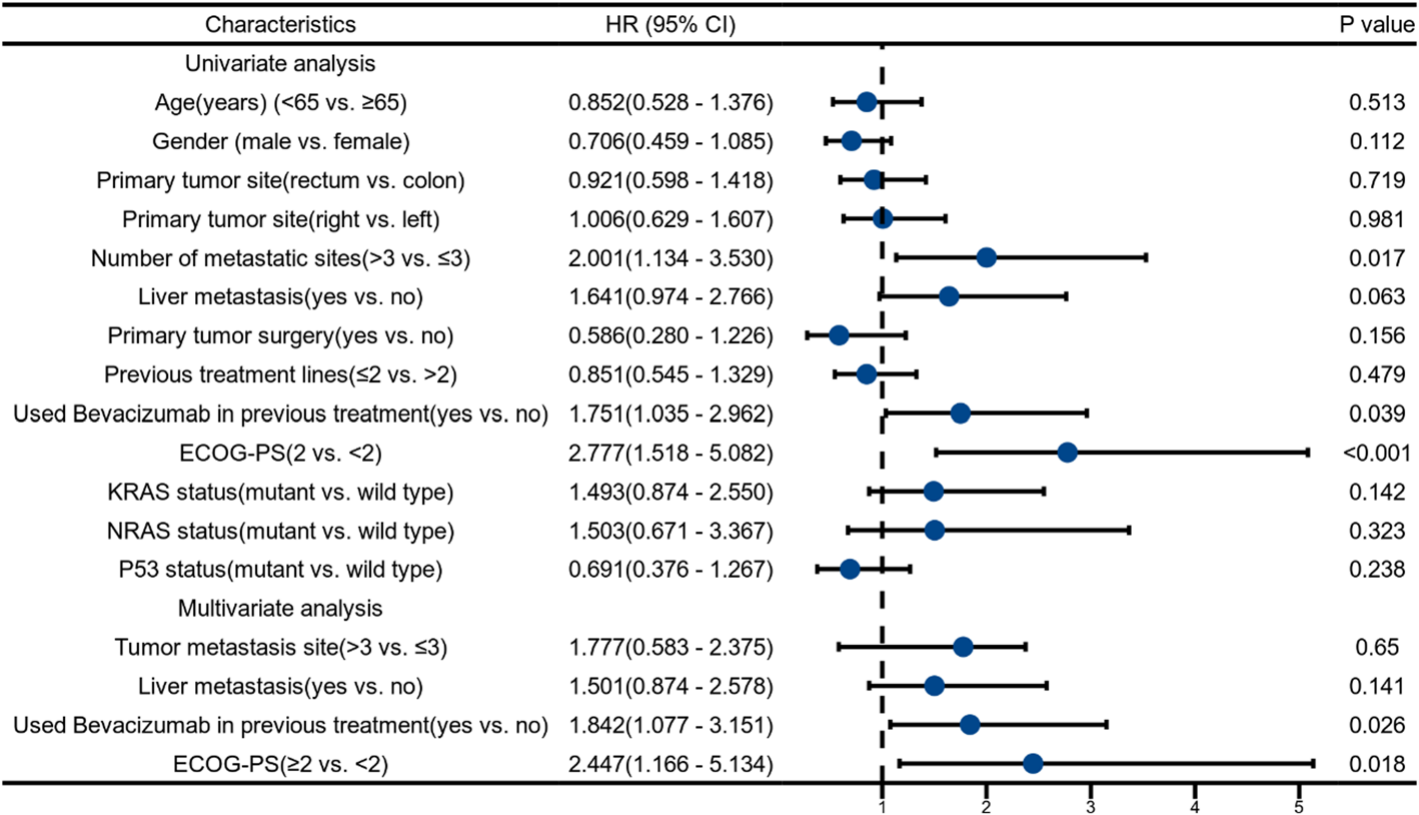
Factors correlated with PFS

**Table 4:**
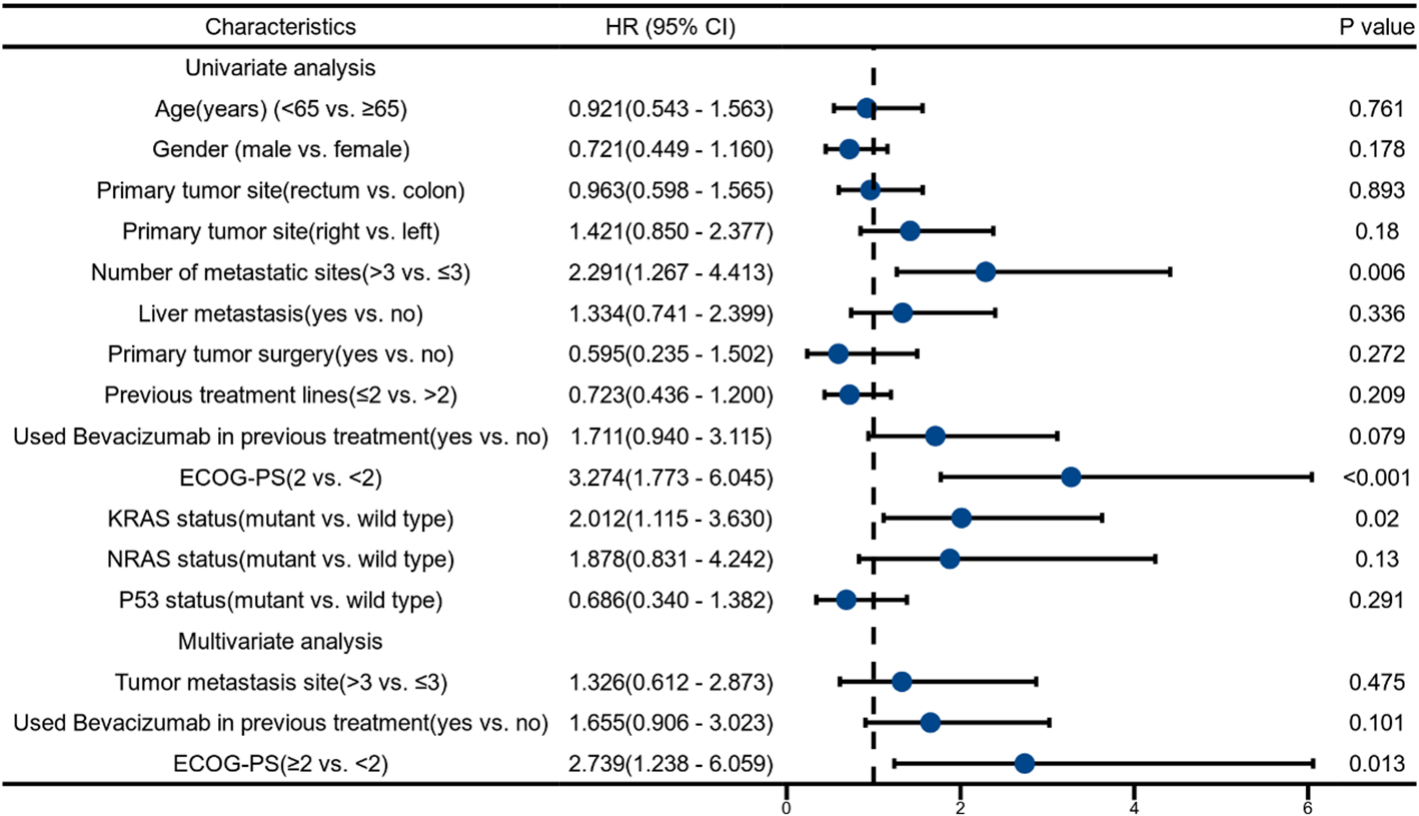
Factors correlated with OS

For OS, univariate analyses identified ≥ 3 metastatic sites (HR, 2.291; 95% CI, 1.267–4.412; *P* = 0.006), ECOG PS = 2 (HR, 3.274; 95% CI, 1.773–6.045; *P* < 0.001), and KRAS mutation (HR, 2.012; 95% CI, 1.115–3.630; *P* = 0.020) as adverse factors (Table 4). In the multivariable model, ECOG PS = 2 remained independently associated with shorter OS (HR, 2.739; 95% CI, 1.238–6.059; *P* = 0.013).

### Safety

Adverse events (AEs) were graded per CTCAE v5.0. Common AEs included neutropenia (63.0%), leukopenia (67.0%), anemia (44.6%), malaise (55.4%), nausea (45.7%), anorexia (31.5%), and diarrhea (23.9%) (Table 5). Grade ≥ 3 events were most frequent for leukopenia (22.8%) and neutropenia (31.5%), whereas most other AEs were grade 1–2. Between-group comparisons using the chi-square test showed lower incidences of most AEs in the bi-weekly group (Table 5), with statistically significant reductions in any‑grade neutropenia, anemia, and malaise (*P* = 0.023, *P* = 0.029, and *P* = 0.034, respectively).

**Table 5:** Comparison of adverse events in patients

Adverse event	Total(*n* = 92) *n*(%)		Bi-weekly(*n* = 21) *n*(%)		Four-weekly(*n* = 71) *n*(%)		*P* value	
	All grades	≥ 3	All grades	≥ 3	All grades	≥ 3	All grades	≥ 3
**Hematologic**								
Neutropenia	58(63.0)	29(31.5)	9(42.9)	4(19)	48(67.6)	25(35.2)	**0.023**	0.161
Leukopenia	62(67.4)	21(22.8)	12(57.1)	4(19)	50(70.4)	17(23.9)	0.254	0.862
Anemia	41(44.6)	4(4.3)	5(23.8)	0(0.0)	36(50.7)	4(5.6)	**0.029**	0.570
Decreased platelet	19(20.7)	6(6.5)	2(9.5)	1(4.8)	17(23.9)	5(7.0)	0.260	1.000
Hypoalbuminemia	17(18.5)	0(0.0)	3(14.3)	0(0.0)	14(19.7)	0(0.0)	0.808	1.000
Elevated ALT/AST	16(17.4)	1(1.1)	3(14.3)	0(0.0)	13(18.3)	1(1.4)	0.921	1.000
Elevated creatinine	10(10.9)	1(1.1)	2(9.5)	0(0.0)	8(11.3)	1(1.4)	1.000	1.000
Elevated bilirubin	8(8.7)	0(0.0)	2(9.5)	0(0.0)	6(8.5)	0(0.0)	1.000	1.000
Febrile neutropenia	2(2.2)	2(2.2)	0(0.0)	0(0.0)	2(2.8)	2(2.8)	1.000	1.000
**Non-hematologic**								
Fatigue	51(55.4)	1(1.1)	9(42.9)	0(0)	42(59.2)	1(1.4)	**0.034**	1.000
Nausea	42(45.7)	3(3.3)	8(38.1)	1(4.8)	34(47.9)	2(2.8)	0.429	0.545
Anorexia	29(31.5)	0(0.0)	6(28.6)	0(0.0)	20(28.2)	0(0.0)	0.971	1.000
Diarrhea	22(23.9)	2(2.2)	3(14.3)	0(0.0)	19(26.8)	2(2.8)	0.239	1.000
Hypertension	18(19.6)	1(1.1)	5(23.8)	0(0.0)	13(18.3)	1(1.4)	0.806	1.000
Proteinuria	16(17.4)	0(0.0)	3(14.3)	0(0.0)	13(18.3)	0(0.0)	0.921	1.000
Vomiting	14(15.2)	1(1.1)	2(9.5)	0(0.0)	12(16.9)	1(1.4)	0.630	1.000

## Discussion

TAS-102 plus bevacizumab over TAS-102 monotherapy, and guidelines prioritize the combination. Our outcomes were comparable to C-TASK FORCE (16-week PFS 42.9%) [19] and within the ranges reported across several small retrospective/phase II series [21–25], though somewhat less favorable than SUNLIGHT (mPFS 5.6 months, 95% CI 4.5–5.9; mOS 10.8 months, 95% CI 9.4–11.8) [26]. Differences likely reflect case-mix: our ECOG-PS distribution included PS = 2 (25.0%/60.9%/14.1% for PS = 0/1/2), whereas SUNLIGHT enrolled only PS = 0–1 (48.4%/51.6%) and excluded PS = 2. Ethnic variability in drug sensitivity and use of clinical versus radiographic progression may also contribute.

Genomic context may also influence outcomes. Mechanistic work suggests that RAS and P53 alterations affect mCRC prognosis [27–29], but clinical findings are heterogeneous. Some studies reported shorter mPFS with mutant RAS in patients treated with TAS-102 plus bevacizumab (5.4 vs. 4.0 months; *P* = 0.03) [23], others associated mutant RAS with worse mPFS and mOS [19, 30], whereas another found no association [24]. In our analysis, KRAS mutation was associated with inferior OS (KRAS-wt vs. KRAS-mut: 11.8 vs. 7.7 months; *P* = 0.018), which may help reconcile divergent prior results due to varying patient compositions. However, some reports suggest diminished benefit is largely driven by KRAS G12 variants instead of all KRAS variants [31]. Given the heterogeneity of KRAS mutations, future large-scale studies targeting specific KRAS subtypes will be necessary. By contrast, P53-mut mCRC did not demonstrate the anticipated adverse prognostic impact in our cohort. Different P53 mutation types can confer distinct prognoses. For example, amino-terminal mutations have been linked to better outcomes than wild-type in certain contexts, potentially reflecting greater treatment sensitivity [27]. Moreover, FGFR4 p.G388R has been proposed as an emerging biomarker for TAS-102 response in mCRC. In a real-world study, the FGFR4 p.G388R was enriched among patients with durable disease control and associated with longer survival, suggesting a predictive signal [32]. Prospective studies incorporating FGFR4 genotyping, alongside variant-level RAS profiling, could refine risk stratification and optimize TAS-102-based regimens.

Beyond genomics, established prognostic factors remain relevant [33–35]. ECOG-PS = 2 independently predicted shorter PFS (HR, 2.447; 95% CI, 1.166–5.341; *P* = 0.018) and OS (HR, 2.739; 95% CI, 1.238–6.059; *P* = 0.013). Prior bevacizumab exposure was associated with shorter PFS (HR, 1.842; 95% CI, 1.077–3.151; *P* = 0.026) but not OS (HR, 1.711; 95% CI, 0.940–3.115; *P* = 0.079), suggesting possible partial resistance without a clear survival detriment. Patients with ≥ 3 metastatic sites were adverse in univariate analyses.

Safety findings were in line with expectations for this combination. Hematologic AEs were common but largely manageable. Neutropenia occurred in 63.0% (all grades) and 31.5% (grade ≥ 3), with a lower grade ≥ 3 rate than in SUNLIGHT (43.1%) despite similar all-grade incidence (62.2%) [26], potentially reflecting proactive granulocyte-stimulating support, which aligns with guidance discouraging dose reduction in uncomplicated neutropenia while recommending supportive measures [36]. Other AEs appeared more frequent than in SUNLIGHT but were predominantly low grade and manageable [26]. Comparing schedules, the bi-weekly regimen was associated with lower rates of most AEs, notably neutropenia (42.9% vs. 67.6%) and anemia (23.8% vs. 50.7%), with generally reduced non-hematologic toxicities; hypertension and anorexia were exceptions. Efficacy was comparable, with no significant differences in mPFS or mOS. Overall, the bi-weekly approach may offer improved tolerability without compromising disease control.

As a retrospective, real-world study, it has inherent limitations including selection bias and unmeasured confounding factors which cannot be fully adjusted for. For example, regimen selection was non-random and reflected evolving local practice after emerging evidence suggested improved tolerability with bi-weekly administration; physician-patient shared decision-making considered baseline hematologic parameters, history of neutropenia, comorbidities, concomitant medications, travel constraints, and treatment adherence. The marked group-size imbalance (bi-weekly *n* = 21 vs. four-weekly *n* = 71) limits the robustness of cross-schedule comparisons. For efficacy analyses, we required completion of at least two cycles to ensure adequate exposure and at least one assessment; however, this criterion may introduce survivorship bias and risk overestimating benefit and tolerability. Moreover, molecular data were missing for some cases due to exhaustion of archival tissue, loss of test reports, or lack of testing because of patients’ financial constraints, the sample sizes for analyzing specific biomarkers (e.g., NRAS, *n* = 7) were also insufficient, thereby limiting the statistical power and precision of the study. Accordingly, all comparative and biomarker findings should be viewed as exploratory and hypothesis-generating.

## Conclusion

In this retrospective, real-world study, the two regimens demonstrated comparable disease control, and the bi-weekly regimen appeared to be better tolerated, representing a reasonable potential alternative. Nevertheless, these findings should be interpreted as exploratory, and future prospective studies are warranted.

## Data Availability

The datasets generated and/or analyzed during the current study are available from the corresponding author on reasonable request.
